# The Roles of Ebola Virus Soluble Glycoprotein in Replication, Pathogenesis, and Countermeasure Development

**DOI:** 10.3390/v11110999

**Published:** 2019-10-31

**Authors:** Wenjun Zhu, Logan Banadyga, Karla Emeterio, Gary Wong, Xiangguo Qiu

**Affiliations:** 1 National Microbiology Laboratory, Public Health Agency of Canada, Winnipeg, MB R3E 3R2, Canada; karla.emeterio@canada.ca (K.E.); xiangguo.qiu@canada.ca (X.Q.); 2 Department of Medical Microbiology and Infectious Diseases, Max Rady College of Medicine, University of Manitoba, Winnipeg, MB R3E 0J9, Canada; 3 Institute Pasteur of Shanghai, Chinese Academy of Sciences, Shanghai, China; garyckwong@hotmail.com; 4 Département de microbiologie-infectiologie et d’immunologie, Université Laval, Québec, QC G1V 0A6, Canada

**Keywords:** filovirus, Ebola virus, glycoprotein, GP, sGP, pathogenesis

## Abstract

Ebola virus (EBOV) is a highly lethal pathogen that has caused several outbreaks of severe hemorrhagic fever in humans since its emergence in 1976. The EBOV glycoprotein (GP_1,2_) is the sole viral envelope protein and a major component of immunogenicity; it is encoded by the *GP* gene along with two truncated versions: soluble GP (sGP) and small soluble GP (ssGP). sGP is, in fact, the primary product of the *GP* gene, and it is secreted in abundance during EBOV infection. Since sGP shares large portions of its sequence with GP_1,2_, it has been hypothesized that sGP may subvert the host immune response by inducing antibodies against sGP rather than GP_1,2_. Several reports have shown that sGP plays multiple roles that contribute to the complex pathogenesis of EBOV. In this review, we focus on sGP and discuss its possible roles with regards to the pathogenesis of EBOV and the development of specific antiviral drugs.

## 1. Introduction

Filoviruses are negative stranded, nonsegmented RNA viruses that belong to the family *Filoviridae*, which is further subdivided into five genera: *Marburgvirus*, *Ebolavirus*, *Cuevavirus, Striavirus,* and *Thamnovirus* [1]. The *Ebolavirus* genus consists of five viruses representing five distinct species: Ebola virus (EBOV; species *Zaire ebolavirus*), Sudan virus (SUDV; *Sudan ebolavirus*), Bundibugyo virus (BDBV; species *Bundibugyo ebolavirus*), Taï Forest virus (TAFV; species *Taï Forest ebolavirus*), and Reston virus (RESTV; species *Reston ebolavirus*). The recently described Bombali virus (BOMV) has been proposed to belong to a sixth species, tentatively called *Bombali ebolavirus* [1,2]. EBOV attracted global attention following a large outbreak in West Africa during 2013–2016, which led to over 10,000 deaths [3]. EBOV causes severe hemorrhagic fever in humans and non-human primates (NHPs), with a fatality rate up to 90% [3,4]. Infected patients typically develop fever, headache, vomiting and diarrhea, progressing to shock and multiorgan failure in severe cases. Hemorrhage is also observed in a subset of victims. Neurological symptoms such as meningoencephalitis, seizures, and coma have also been reported [5,6,7]. Bats are suspected to be the reservoir of EBOV, and introduction into humans is thought to occur following direct contact with bats or bat excreta or through contact with other susceptible animals, such as NHPs [3]. Direct transmission between humans is the major mode of EBOV spread because the virus can be shed in many bodily fluids, including blood, saliva, tears, urine, semen, and sweat [3].

**Figure 1:**
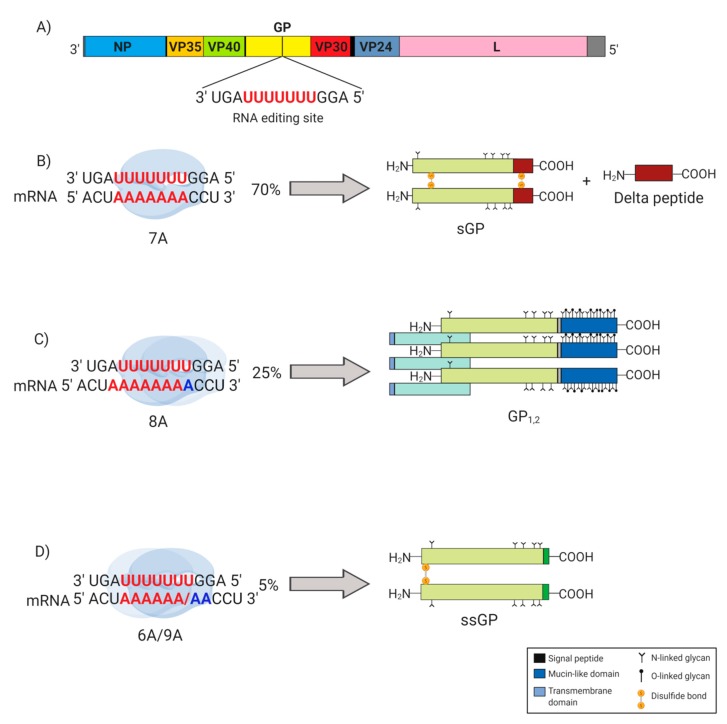
Glycoprotein (GP) gene editing in Ebola virus (EBOV). Highlighted (in red) in the EBOV *GP* gene are seven consecutive uridine (U) residues that act as the transcriptional editing site. (**A**) The EBOV genome encodes seven structural proteins, including the nucleoprotein (NP), virion protein (VP) 35, VP40, glycoprotein (GP_1,2_), VP30, VP24, and the RNA-dependent RNA polymerase (L). (**B**) The majority (~70%) of transcripts produced are unedited, containing seven adenosine (7A) residues, and translated to produce soluble glycoprotein (sGP), which yields delta (Δ)-peptide upon proteolytic cleavage. (**C**) Approximately 25% of the transcripts contain eight adenosine (8A) residues due to the addition of a non-templated A (in blue) added by the RNA polymerase as it stutters at the editing site. The non-templated A results in a frameshift that extends the length of the open reading frame, giving rise to the surface glycoprotein (GP_1,2_). (**D**) For approximately 5% of the total transcripts, RNA polymerase stuttering at the editing site results in mRNA transcripts containing either six adenosine (6A) or nine adenosine (9A) residues, both of which encoding the small soluble glycoprotein (ssGP).

The EBOV genome is approximately 19 kb in size, and it encodes seven structural proteins, including the nucleoprotein (NP), virion protein (VP) 35, VP40, glycoprotein (GP_1,2_), VP30, VP24, and the RNA-dependent RNA polymerase (L) (Figure 1A) [4,8]. NP, which binds to the viral genome, is the major component of the nucleocapsid, along with VP35 and VP24. VP30 is also a component of the nucleocapsid, in addition to its role as a transcription factor. VP40 is the matrix protein, which drives the formation of new virus particles, and L, along with VP35 as a polymerase cofactor, facilitates genome replication and transcription. GP_1,2_, a class I membrane protein residing in the viral envelope, serves as the major viral attachment and entry factor, although the complex mechanisms governing EBOV entry into the host cell are only partially understood [9]. Intriguingly, through a co-transcriptional editing mechanism, the *GP* gene of all ebolaviruses encodes two additional, nonstructural proteins known as soluble GP (sGP) and small soluble GP (ssGP) (Figure 1) [10,11,12]. Both sGP and ssGP have been proposed to play a number of different roles during infection [10,13,14,15,16,17]. This review presents our current understanding of sGP, and it describes the roles of this protein during the various phases of EBOV pathogenesis, with the view of connecting structure to function and ultimately informing the rational development of antiviral therapies.

## 2. EBOV *GP* Gene Products

The *GP* gene encodes three proteins, GP_1,2_, sGP, and ssGP, that are the product of a unique co-transcriptional editing strategy (Figure 1) [10,11,12,18]. sGP mRNA is the primary product of the *GP* gene, accounting for approximately 70% of transcripts [10,11]. Occasionally, however, the viral polymerase stutters during transcription at a stretch of seven uridines present in the viral genome just upstream of the sGP stop codon. This stuttering results in the addition of a non-templated adenosine residue in the nascent transcript, thus producing a frame shift that alters the position of the stop codon. The resulting longer mRNA, which accounts for approximately 25% of the transcripts derived from the GP gene, encodes the GP_1,2_ precursor [10,11]. The co-transcriptional addition of two adenosine residues, or the subtraction of a single adenosine, results in a different frame shift that produces a much shorter transcript, which encodes ssGP [10]. Thus, transcripts that encode sGP contain a stretch of seven adenosine residues (7A), transcripts that encode GP_1,2_ contain a stretch of eight adenosine residues (8A), and transcripts that encode ssGP contain a stretch of six or nine adenosine residues (6A/9A) [11,12]. Notably, while this co-transcriptional strategy is employed by all ebolaviruses and cuevaviruses, it is not employed by the marburgviruses, including Marburg virus (MARV) and Ravn virus, which consequently do not produce sGP or ssGP [19]. 

**Figure 2:**
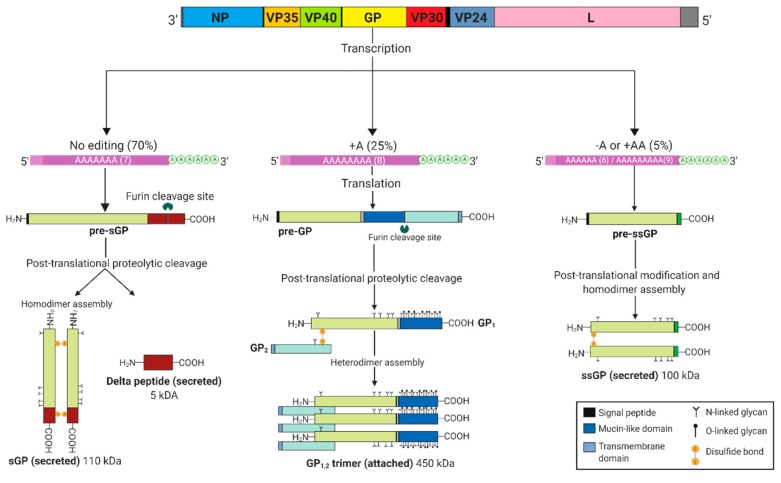
Ebolavirus *GP* gene products. Co-transcriptional editing at the *GP* gene editing site results in three major transcripts that are afterwards translated to various glycoprotein products, initially synthesized as pre-sGP, pre-GP and pre-ssGP. Post-translational cleavage by furin at the carboxy terminus of pre-sGP generates a 5 kDa delta (Δ)-peptide and an sGP monomer that further assembles into a 110 kDa homodimer linked by two disulfide bonds. Similarly, pre-GP (also known as GP_0_) undergoes a proteolytic cleavage resulting in the formation of a disulfide linked GP_1,2_ heterodimer that trimerizes into a 450 kDa viral surface glycoprotein. Pre-ssGP does not undergo post-translational cleavage, but likewise homodimerizes via formation of a single disulfide bond. Additionally, all the glycoproteins are *N*-glycosylated, with GP_1,2_ experiencing further O-glycosylation.

Following transcription, GP_1,2_ is produced as a precursor protein, known as pre-GP or GP_0_, which is then cleaved post-translationally by furin-like proteases to yield the ectodomains GP_1_ and GP_2_ (Figure 2) [9,20,21]. GP_1_ and GP_2_ then form dimers, which in turn form trimers to produce the mature and functional heterotrimeric GP_1,2_. As the sole viral protein expressed on the surface of the virion envelope, GP_1,2_ facilitates attachment to host cells by interacting with a variety of cell surface factors, including carbohydrate-binding receptors [9]. Following internalization of the virion by macropinocytosis and progression through the endocytic pathways, cleavage of the GP_1,2_ mucin-like domain and glycan cap by cathepsins L and B reveals the receptor binding site. Interaction between the GP_1,2_ receptor binding site and the filovirus receptor Niemann–Pick C1 (NPC1) in the late endosome/early lysosome, results in a conformational change in GP_1,2_ that drives fusion of the virion and endosomal membranes, resulting in the release of the virus genome into the cytoplasm [22]. Interestingly, proteolytic cleavage of surface-expressed GP_1,2_ by the cellular tumor necrosis factor α converting enzyme (TACE) removes the transmembrane anchor and liberates GP_1_ in complex with a truncated GP_2_, a product known as shed GP [23]. Shed GP has been reported to activate the Toll-like receptor (TLR) 4 signaling pathway, resulting in the expression of pro-inflammatory cytokines, maturation of macrophages and dendritic cells [24]. Shed GP was also demonstrated to increase endothelial permeability and block the activity of neutralizing antibodies [23,25].

sGP, the most abundant product from the *GP* gene, is initially synthesized as a precursor protein known as pre-sGP (Figure 2) [26]. Subsequent post-translational proteolytic cleavage by furin and dimerization produces mature sGP, which is secreted from cells. The byproduct of furin cleavage at amino acid position 324 in the C-terminus of sGP produces the Δ-peptide, which is also secreted from cells [26]. Although sGP shares the first 295 N-terminal amino acids with GP_1,2_, the structure and function are markedly different: sGP forms 110 kDa homodimers in a parallel orientation, whereas GP forms anti-parallel trimers [10,27]. Since sGP is the primary product of the *GP* gene and shares common residues with GP_1,2_, it may play a role in the replication cycle and pathogenesis of EBOV. sGP may also be an excellent biomarker for specific diagnosis of EBOV since it is secreted in abundance into the blood during the early stages of infection. Moreover, sGP constitutes a promising target for vaccines and antiviral therapeutics due to its many functions on the host immune system. 

The third product of the *GP* gene, ssGP, has been shown to be secreted as a 100 kDa homodimer [10]; however, its roles during EBOV infection are still not clearly defined (Figure 2).

## 3. sGP May Substitute as a Structural Protein

sGP, which shares the same N-terminus as GP_1,2_, is commonly thought of as a nonstructural, secretory glycoprotein. Both sGP and GP_1_ share a cysteine residue at position 53, which forms a disulfide bond with cysteine 609 in GP_2_ [12]. Data suggest that sGP can substitute for GP_1_, forming a complex with GP_2_ to create a functional glycoprotein [13]. Indeed, vesicular stomatitis virus (VSV) pseudotyped with the sGP–GP_2_ complex resulted in an infectious virus. However, sGP was also shown to result in a reduction in virus titer when overexpressed with GP_1,2_. Together, these data suggest not only that sGP may have a novel role as a structural protein by replacing GP_1_, but they also imply that sGP may have a role in limiting the cytotoxicity of GP_1,2_. The GP_1_ mucin-like domain is known to have cytotoxic effects, and it has been previously reported that a recombinant EBOV encoding only GP_1,2_ and not sGP showed significantly increased cytotoxicity [14]. It is therefore tempting to speculate that by replacing a certain amount of GP_1_, sGP may decrease the overall cytotoxicity of EBOV to the host cells and, in so doing, facilitate more efficient replication and promote infectivity.

## 4. sGP Serves as a Virulence Factor

Previous studies have shown that a recombinant virus encoding eight uridines (8U) at the *GP* transcriptional editing site (producing an 8A transcript) quickly reverts to the wild type 7U (7A transcript) in guinea pigs [28]. This finding suggests that there is a strong selective pressure in vivo for sGP production and that sGP plays a critical role during EBOV infection. Moreover, passage of EBOV in Vero E6 cells quickly leads to an eight uridine genotype, whereas passage in Huh7 cells favors the seven uridine genotype [29], indicating that sGP expression may also be cell type dependent. Indeed, although recombinant EBOV devoid of sGP exhibited increased synthesis of GP_1,2_ and enhanced cytotoxicity [14], the virus proved less pathogenic in animals [15]. Guinea pigs infected with sGP-deficient EBOV had lower viral loads throughout the course of the experiments compared to animals infected with wild type EBOV, although both groups developed an antibody response against GP [15]. This finding suggests that sGP may play a role in the pathogenicity of EBOV, and it provides evidence supporting the idea that GP_1,2_ cytotoxicity may limit virus spread. Conversely, however, another study found that the absence of sGP did not influence the infectivity of a recombinant, sGP-deficient EBOV, as this virus did not show significant attenuation in guinea pigs [30]. The reason for the discrepancy between these two studies remains unclear, although it may berelated to the differences in the recombinant viruses used or differences in the outbred guinea pigs. Further studies will be required to investigate the effect of sGP on EBOV virulence in other animal models, such as NHPs, which more closely resemble humans. 

Interestingly, Whitmer et al. investigated persistent infections in EBOV survivors and sequenced EBOV from semen samples [31]. In virus sequences obtained from one patient, the authors found a cytidine insertion at the *GP* editing site that resulted in the genome sequence encoding a full-length GP_1,2_ with the frequency of 34%–65%. This is the first published case from a human patient to show that selective pressure from specific tissue compartments, such as immune privileged sites, may cause mutations that decrease sGP production [31]. However, whether this insertion mutation resulted in a significant decrease in sGP production, and whether this might have contributed to patient survival or virus persistence, remains unclear.

## 5. sGP Alters the Immune Response

Previous studies have suggested that sGP may exhibit anti-inflammatory activities [17]. Kindzelskii et al. showed that sGP dramatically reduces the amount of CD16b receptor on human neutrophils in a dose-dependent manner [32]. They also found that sGP could induce a conformational change in this receptor, helping prevent the activation of neutrophils and stunt the innate immune response by preventing immune complexes to activate neutrophil metabolic flux [32]. Indeed, during EBOV infection, many areas of focal tissue destruction in multiple organs, including the liver and kidneys, lack leukocyte infiltration, although neutrophil aggregation can still be observed within the vascular system [17]. This may be explained by the inhibition of the transmigration process of leukocytes through the endothelium due to the anti-inflammatory effect of sGP. Wahl-Jensen et al. showed that VP40 and GP_1,2_ were able to activate endothelial cells and decrease barrier function—which was enhanced by the cytokine tumor necrosis factor alpha (TNF-α)—while sGP induced a recovery of endothelial barrier function following treatment with TNF-α [17]. Thus, sGP seems to play an anti-inflammatory role by protecting the integrity of the endothelium and promoting the recovery of barrier function, which may support virus replication. sGP was also shown to reduce the production of pro-inflammatory, but not anti-inflammatory, cytokines by macrophages [33]. Moreover, although sGP was shown not to affect phagocytosis, it significantly diminished the chemotaxis of activated macrophages [33]. Together, these data suggest that sGP impairs effector activities of immune cells before they get infected, thereby creating a pool of susceptible macrophages primed for infection [33]. The preservation of the phagocytic ability of macrophages also ensures that these cells can continue to take up virions and promote cell infection [33]. Indeed, the anti-inflammatory functions of sGP may provide a relatively stable environment for EBOV-infected cells and support virus replication and transmission to other cells. More studies will be needed to address the mechanism of the potential anti-inflammatory function of sGP, and whether the effect is specific for TNF-α or other components in the signaling pathway. It would also be interesting to determine whether the sGP functions observed in cell culture are retained in vivo, and whether eliminating sGP might help restore the normal function of immune cells.

## 6. sGP Is an Important Target for the Immune Response

sGP has been shown to act as a decoy antigen through binding specific antibodies against GP_1,2_, possibly resulting in antigenic subversion and contributing to systemic viral spread in the host [34]. High amounts of sGP are thought to divert the host humoral immune response away from GP_1,2_ and towards sGP, a phenomenon that is supported by data showing that sGP is able to efficiently compete for anti-GP_1,2_ antibodies from mice immunized by sGP [34]. Additionally, sGP can interfere with viral neutralization by antisera from mice immunized with GP_1,2_ and sGP [34]. However, when mice were immunized with GP_1,2_ alone (in which expression of sGP was disabled), anti-GP_1,2_ antibodies showed low cross-reactivity with sGP, consistent with previous studies demonstrating that antibodies generated in response to GP_1,2_ do not share many epitopes with sGP [35,36]. One recent study showed that a chimeric EBOV expressing BDBV GP but not sGP, displayed increased susceptibility to a monoclonal antibody isolated from a survivor of BDBV infection, further demonstrating a role for sGP in evasion of antibody neutralization [37]. 

Somewhat paradoxically, Liu et al. recently showed that intradermal or intramuscular immunization with vaccines containing the sGP subunit plus adjuvant can confer effective protection to mice against lethal EBOV challenge by producing antibodies that can neutralize both GP_1,2_ and sGP [38]. These data demonstrate that sGP elicits sGP/GP_1,2_ cross-reactive antibodies, and they may suggest that neutralization of sGP itself offers some therapeutic benefit. Indeed, recent analysis of a number of different sGP/GP_1,2_ cross-reactive antibodies demonstrated that some of these antibodies could more effectively neutralize wild type EBOV (which expresses sGP) than VSV pseudotyped with EBOV GP (which does not express sGP) [39]. This study therefore raises the intriguing possibility that certain sGP/GP_1,2_ cross-reactive antibodies may depend on an interaction with sGP in order to achieve their therapeutic benefit. Notably, 13C6, a monoclonal antibody that is part of the ZMapp cocktail, has been shown to bind both sGP and GP_1,2_ [16,40], and it is tempting to speculate that binding to sGP may be critical for this antibody’s therapeutic function. Moreover, analysis of the immune effector functions of GP_1,2_- and sGP-specific IgA1 antibodies isolated from human survivors revealed an association with antibody-dependent phagocytosis by neutrophils (ADNP) [41], demonstrating the important role an immune response against sGP may play during EBOV infection.

With respect to the cellular immune response, a recent study investigated the EBOV-specific T-cell memory responses in patients infected during the 2013-2016 EBOV outbreak [42]. The study revealed that 8/10 individuals had T-cell responses to both GP_1,2_ and sGP in their peripheral blood mononuclear cells (PBMCs), while only 1/10 responded to GP_1,2_ but not sGP. These data therefore suggest that the portion of GP_1_ shared with sGP may be more immunogenic than GP_1,2_ in inducing an effector T-cell response, thereby diverting the immune response away from GP_1,2_ [42].

## 7. Δ-Peptide May Have a Role in Viral Replication and Pathogenicity

Cleavage of the sGP protein by furin yields Δ-peptide, a 40 aa carboxy-terminal fragment that is released from cells more slowly and at lower amounts than sGP [26,43]. The function of the Δ-peptide is still unknown, but sequence analysis suggests it could be a candidate viroporin, capable of permeabilizing cellular membranes. Δ-peptide possesses a motif similar to one found in known viroporins, such as rotavirus NSP4 and HIV LLP-1, suggesting that this peptide may act as a lysin or cytotoxin [26]. Indeed, a recent study showed that the Δ-peptide has a high abundance of aromatic and cationic residues, which are found in many membrane-permeabilizing peptides [44]. Another study has shown that Δ-peptide is able to permeabilize mammalian cell plasma membranes at micromolar concentrations, so it may alter the permeation of ionic compounds and small molecules into the cells, contributing to viral pathology by causing disruption of cell function and cell death [45]. On the other hand, Δ-peptide may function to modulate virus entry and infection of certain cell types. When Δ-peptide-Fc domain chimeras were added externally to cells, cell viability was not affected; however, the chimeras inhibited the entry of all filoviruses, including MARV [43]. These data suggest that Δ-peptide inhibits GP_1,2_ interaction with cellular attachment factors, and while the implications of this function are unclear, it may help facilitate virion budding or prevent superinfection [43]. 

## 8. The Roles of ssGP in Pathogenesis Remain Unknown

ssGP is the second, nonstructural glycoprotein produced through RNA editing during ebolavirus infection, with less than 5% of *GP* gene transcripts thought to be specific for ssGP mRNA [46]. ssGP possesses the same 295 N-terminal amino acid sequence with GP_1,2_ and sGP, differing at their C-terminal sites [10]. Based on their shared sequence, it was previously postulated that ssGP may function similarly to sGP; however, this appears not to be the case. Unlike sGP, ssGP did not exhibit anti-inflammatory function without effect on the endothelial barrier restoration [10]. This may be explained by the observation that ssGP does not contain the cysteine 306 which sGP did. Moreover, Mehedi et al. investigated the binding of ssGP to peripheral blood mononuclear cells and found that it did not interact with CD16b, suggesting that ssGP does not possess the same anti-inflammatory functions as sGP [10]. Thus, the role of ssGP in ebolavirus pathogenesis remains unclear, and more studies are needed to unravel its potential as an antiviral target. 

## 9. Perspectives on the Role of sGP as a Biomarker for Diagnosis and a Target for Antiviral Therapy

**Figure 3:**
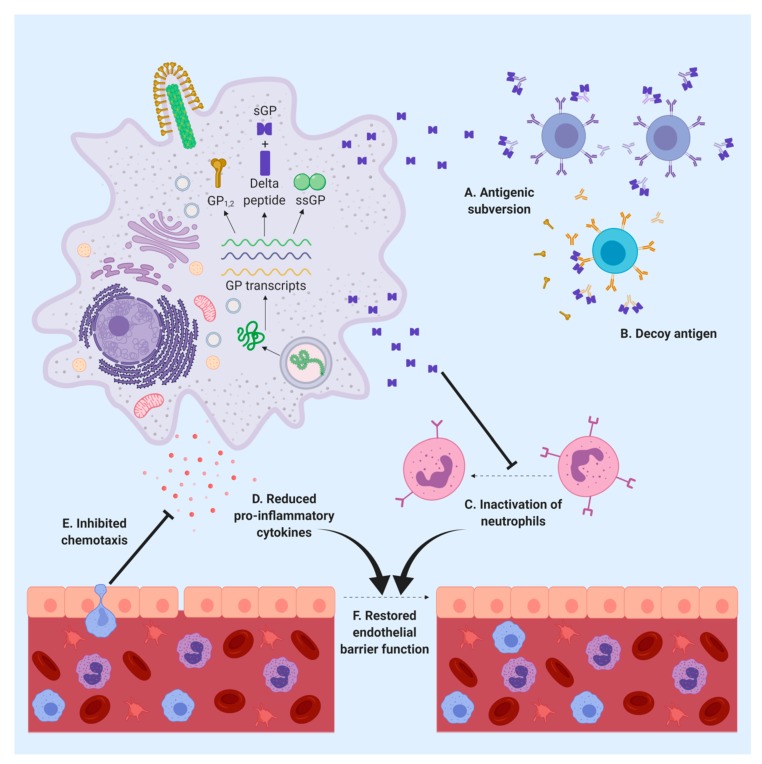
Possible functions of sGP in the pathogenesis of EBOV. Dimeric sGP is secreted into the extracellular space where it may facilitate antigenic subversion (**A**), act as a decoy antigen (**B**), prevent activation of neutrophils (**C**), reduce the production of pro-inflammatory cytokines (**D**), and/or inhibit immune cell chemotaxis (**E**). Consequently, a combination of inactivated neutrophils and reduced levels of pro-inflammatory cytokines may aid in restoring endothelial barrier function (**F**).

At present, the development of post-exposure treatments for Ebola virus disease is mostly focused on neutralizing antibodies against GP_1,2_ [47]. Indeed, one monoclonal antibody, mAb114, and two cocktails of monoclonal antibodies, ZMapp and REGN-EB3, have been evaluated in a randomized clinical trial during the ongoing EBOV outbreak in the Democratic Republic of the Congo [48]. However, as one of the most abundant proteins produced during EBOV infection, sGP may also be a promising target of antiviral therapy. Firstly, sGP may be targeted as part of antibody treatments. Since sGP can bind to GP/sGP cross-reactive antibodies, it may be necessary to use sGP-specific antibodies to diminish the binding of sGP to the treatment antibodies, thereby enhancing their therapeutic effect. Secondly, sGP has shown some effects on immune cells such as macrophages, inhibiting their ability to attack EBOV or remove virus infected cells. Inhibiting these deleterious functions of sGP may interfere with EBOV replication and reduce disease severity. Thirdly, sGP can subvert the host immune system by shifting the antibody response towards sGP-specific antibodies and away from GP-specific, neutralizing antibodies. Thus, it may be a promising therapeutic strategy to administer both sGP-specific antibodies along with sGP/GP_1,2_-cross-reactive antibodies to limit the decoy effect of sGP and enhance neutralization of GP_1,2_. Finally, sGP may act as a biomarker for diagnosis of EVD, since sGP is produced at an early stage of EBOV infection and secreted systemically in high amounts. Indeed, sGP can be detected by ELISA or similar methods, and its detection may aid in early diagnosis of infection. Overall, sGP can be considered a promising target in the treatment of EBOV infection and a key factor in EBOV pathogenesis (Figure 3). 

## 10. Conclusions

The 2013–2016 EBOV outbreak in West Africa caused global concern, particularly in countries with endemic/imported cases [49]. There is an urgent need to design effective therapies to prevent future outbreaks. Due to its multifaceted roles in viral pathogenesis, sGP is a promising target for the diagnosis and treatment of EBOV infections. Several therapeutic antibodies targeting GP have been developed and are currently in clinical trials [47]. In addition to direct roles of GP_1,2_ in the virus replication cycle, sGP and its cleaved product Δ-peptide are all believed to contribute to EBOV infection and replication. However, for all reported functions of sGP, further investigations are required to definitively understand the roles of sGP in EBOV biology and determine whether it can be effectively targeted during infection.
